# A Comparison

**Published:** 1897-10

**Authors:** 


### A COMPARISON.

In doctors' books of two hundred years ago we read the following prescription to ward off fevers: "Take eight pints of rosemary flowers, three pints of shell snails, two handfuls of seed flax, and one puppy nine days old. Wash the snails, kill the dog, fling away the head, and dry the quarters in a linen cloth. Pound all together to a powder, and put the powder into well-corked bottles. It is now ready for use, and if a teaspoonful be taken once a day fever will be kept off."

At the present day the recipe for the same thing is as follows: "Take matter from the heels of a horse that is suffering from 'grease.' Put the matter into the veins of a cow, so that ulcers and running sores are produced. Take lymph from these ulcers; pass it through human subjects; lance the skin of a child, and introduce a particle of the lymph within the skin; then, if a running ulcer ensues, the child will be safe from the form of fever called small-pox for the rest of its life."

Now, I think, there is very little to choose between these two prescriptions in point of absurdity, though there is in harmfulness.—*Fashions of the Day in Medicine and Science, by H. Strickland Constable, page 41.*

